# Supplementary material for: IL-17RA promotes pathologic epithelial inflammation in a mouse model of upper respiratory influenza infection
Source: PLoS Pathog. 2023 Dec 7;19(12):e1011847. doi: 10.1371/journal.ppat.1011847 (PMC10729944; doi:10.1371/journal.ppat.1011847)
Supplement: Attachment — Submitted filename: Letter to Editor-Submission.docx [file ppat.1011847.s001.docx]

Amy L. Hartman, PhD

Academic Editor

PLOS Pathogens

Dear Dr. Hartman,

Thank you for the review of our manuscript “*IL-17RA promotes pathologic epithelial inflammation in a mouse model of upper respiratory influenza infection*” for consideration in PLOS Pathogens. We appreciate the feedback from the editor and an opportunity to revise and re-submit the manuscript. We thank the reviewers for their appreciation as well as their constructive suggestions that have helped improve the manuscript substantially. In light of the reviewers' comments and editor's note, we have performed additional experiments to comprehensively address the concerns raised in original submission. We have answered each query step by step, and the modifications have been highlighted throughout the revised manuscript. The modifications (revisions) have been referred to with a page number and respective line number in the revised manuscript.

We sincerely thank the editor to allow us extensions on the re-submission deadlines to complete the revision work. With the additional data and revisions, we believe to have fully addressed the reviewer's concerns. We hope the revised manuscript is now suitable for publication in PLOS Pathogens.

**Editor comments.**

Dear Professor Khan,

Thank you very much for submitting your manuscript "IL-17RA promotes pathologic epithelial inflammation in a mouse model of upper respiratory influenza infection" for consideration at PLOS Pathogens. As with all papers reviewed by the journal, your manuscript was reviewed by members of the editorial board and by several independent reviewers. In light of the reviews (below this email), we would like to invite the resubmission of a significantly-revised version that takes into account the reviewers' comments.

In particular, the following points need to be addressed experimentally:

- Timing of co-infection: Does prior administration of Spn to WT and KO mice leads to the same level of colonization and inflammation, as raised by reviewer 1?

- The use of ALI at day 7, which contain no ciliated cells, and appear leaky, is unusual and will make it impossible to place the findings of this study in the body of existing data which use mature ALI cultures. It is critical that experiments with ALI are performed with fully mature ALI (day 21), and, preferably, using a human IAV, as pointed out by reviewer 2.

- Determine the kinetics of the IAV infection/inflammation in the NP with one earlier and one later time point in both WT and KO mice, as raised by reviewer 3.

**Author Response.** We performed additional experiments to address three significant (aforementioned) points raised by the editor. The findings from the new experiments is summarized as following:

- Timing of co-infection: Does prior administration of Spn to WT and KO mice leads to the same level of colonization and inflammation, as raised by reviewer 1?

**Author Response.** To address the concern, we colonized WT and IL-17RA KO mice with *Spn* and determined bacterial burden in the nasopharynx 24 hours after establishing the colonization. Our data show that both WT and IL-17RA KO mice had comparable level of *Spn* bacterial density in nasal lavage at 24-hour time point, addressing reviewer’s concern. The new data is referred **as Fig 4C.**

- The use of ALI at day 7, which contain no ciliated cells, and appear leaky, is unusual and will make it impossible to place the findings of this study in the body of existing data which use mature ALI cultures. It is critical that experiments with ALI are performed with fully mature ALI (day 21), and, preferably, using a human IAV, as pointed out by reviewer 2.

**Author Response.** We appreciate the comment. To address the concern, we repeated ALI experiments with differentiated human nasopharyngeal epithelial cells using a human IAV strain (H1N1 pdm09). Our new data show that IL-17A treatment exacerbated IAV-induced pathologic changes in epithelial cells. In particular, our data show that IL-17A treatment synergistically induced cell death and disrupted epithelial barrier integrity of IAV-infected epithelial cells. Epithelial barrier integrity was measured by a combined metric of ZO-I expression, basolateral transfer of FITC-dextran, and *Spn* bacterial translocation from apical to basal chamber of the transwell *via* damaged epithelial monolayer. These new data supported the earlier findings using PR8 influenza virus and infections performed with un-differentiated epithelial cells from 7 day ALI culture. However, for clarity and consistency, we have replaced ALI data (Fig. 4 and 5) of un-differentiated epithelial cells presented in the original submission entirely with the new data sets with differentiated epithelial cells infected with human IAV strain and referred **as Fig 5.**

- Determine the kinetics of the IAV infection/inflammation in the NP with one earlier and one later time point in both WT and KO mice, as raised by reviewer 3.

**Author Response.** To address the concern, we performed H&E staining to determine the differences in nasopharyngeal inflammation/tissue pathology between WT and IL-17RA KO mice as well as IAV viral load on 3, 7, and 14-day post infection (dpi) time points (one earlier and one later time point of 7 dpi). Our new data show that no difference in nasal inflammation was observed between IAV-infected WT and IL-17RA KO mice on 3 dpi. However, WT mice (compared to IL-17RA KO) had significantly higher tissue inflammation at 7 dpi. Both groups (WT and IL-17RA KO) effectively resolved inflammation by 14 dpi. WT mice (compared to KO) had a higher viral load in the NP lavage on 3 and 7 dpi. However, no detectable viral load was found in the nasal lavage of WT and KO mice by 14 dpi. These data suggest that IL-17RA induced pathologic effect is largely limited during the peak inflammation (7 dpi), and the presence of IL-17RA doesn’t produce long-term defects in the resolution of inflammation or IAV viral control in WT mice. The new data is referred **as Fig 2.**

Reviewer's Responses to Questions

Part I - Summary

Please use this section to discuss strengths/weaknesses of study, novelty/significance, general execution and scholarship.

Reviewer #1: In this manuscript, Tripathi et al. interrogated the role of the interleukin-17A (IL-17A) and IL-17 receptor (IL-17RA) signaling in host-defense against two human pathogens, influenza A virus (IAV) and the bacterium Streptococcus pneumoniae. The authors combined in vitro and in vivo infection models to study how loss of IL-17RA impacts the immune response and tissue damage in the upper respiratory tract upon the infection with IAV. The authors demonstrated that IL-17RA knockout mice have decreased recruitment of neutrophils, inflammation, and tissue pathology upon viral infection. They also found that IL-17RA knockout mice were more resistant to S. pneumoniae during coinfection with IAV than WT mice. Finally, the authors established that the addition of IL-17A to IAV infected epithelial cells increased inflammation and cellular pathology and disrupted barrier function. Overall, the manuscript is well-written, and the conclusions are justified by the data. There are a few comments for the author’s consideration.

Reviewer #2: In this study (PPATHOGENS-D-23-00456), Tripathi and colleagues examine the role of IL-17RA as a mediator of host defense and tissue damage in the upper respiratory tract during an influenza virus infection. They convincingly show, in the mouse model, that IL-17RA is a significant regulator of neutrophilia, promotes inflammation in the nasopharynx and promotes Spn colonization to invasive infection. Furthermore, the authors used an in vitro ALI model to examine the impact of IL-17A treatment although the relevance of this chosen model system with non-ciliated human cells and a mouse adapted IAV needs to be clarified. The paper is well written and comprehensible. The dual role of IL-17RA is very interesting and examining it in the mucosal site is very relevant to IAV infection. A better understanding of how influenza virus infection potentiates bacterial secondary infection is important as these are quite common.

Reviewer #3: The study from Tripathi et al. focuses on the role of IL-17RA in the context of IAV infection and coinfection with S. pneumoniae. This study is a follow-up work where they previously had shown the role of IL17 being important during Spn-IAV coinfection. Using a murine model of NP infection combined with transcriptomics and an in vitro cell culture model, they show that IL17RA signaling causes epithelial damage, neutrophil influx, increased viral titers, and increased Spn dissemination to distal sites. The study is well put together, but the reviewer has some concerns with the investigation.

**Author Response.** Thank you for the constructive critique of the manuscript.

**Part II – Major Issues: Key Experiments Required for Acceptance**

Please use this section to detail the key new experiments or modifications of existing experiments that should be absolutely required to validate study conclusions.

Generally, there should be no more than 3 such required experiments or major modifications for a "Major Revision" recommendation. If more than 3 experiments are necessary to validate the study conclusions, then you are encouraged to recommend "Reject".

**Reviewer #1:**

**1.** In figure 3A the data presentation for Spn in the blood is a little confusing. Are the data points that seem to be sitting on the x-axis really zero or are they a substantially higher value that cannot be deciphered because of the scale chosen for the y-axis? Perhaps a log scale would be more appropriate or log-normalized data? This way the reader could better assess the data.

**Author Response.** Thank you for the suggestion. The data sitting on the X-axis are 0 or no bacteria detected in the blood. As suggested, the scale has been re-worked and presented as the log axis. The revised figure is now presented as Fig 4A.

**2.** Have the authors checked to see that prior administration of Spn to WT and KO mice leads to the same level of colonization after 24 hours? Do KO mice just have less bacteria to begin with, prior to administration of IAV? This seems to be an important control that was not considered.

**Author Response.** Thank you for the crucial comment. To address the concern, we colonized WT and IL-17RA KO mice with *Spn* and compared bacterial burden in the nasopharynx 24 hours after establishing the colonization. Our new data show that both WT and IL-17RA KO mice had comparable level of *Spn* bacterial density in nasal lavage at 24 hour time point, addressing reviewer’s key concern. The new data is being presented as Fig. 4C.

The manuscript has been revised to add the findings from the new experiments. The revision refers to the **result** (page 9, line 211-215), **method** (page 16, line 379-383), and **legends** (page 29-30, line 677-680).

**Reviewer #2:**

**1.** The authors state that the respiratory tract is primarily lined by pseudostratified ciliated cells. They show that at D21 the ALI cultures have more ciliated cells than D7, which would be more physiological. What is the rational for using D7, if D21 are more relevant? Also, the values of ZO-1 in mock at D7 in Fig 4E and 5C are quite different. Can the authors explain this?

**Author Response.** We appreciate this highly significant comment from the reviewer. While we believe that experiments with un-differentiated epithelial cells (non-ciliated) from day 7 ALI culture are still relevant, we agree with the reviewer’s suggestion that in this particular context, it would be more appropriate to use fully differentiated epithelial cells expressing cilia and mucus from D21 of ALI culture. Therefore, to address reviewer’s concern, we repeated ALI experiments with differentiated epithelial cells using a human IAV strain (H1N1 pdm09). We have replaced ALI epithelial data presented under the original submission (Fig. 4 and 5) entirely with new data sets with differentiated human nasopharyngeal epithelial cells (21-day ALI culture) infected with a human IAV strain. The new data is referred **as Fig. 5**. The key findings from our new ALI epithelial experiments are summarized as following:

While IAV infection and rIL-17A treatment significantly induced cell death and disrupted ZO-1 and epithelial barrier integrity, rIL-17A treatment severely exacerbated pathologic changes in IAV-infected epithelial cells. In particular, our data show that rIL-17A treatment induced overwhelming cell death (Annexin-V^+^) and disrupted epithelial barrier integrity. Epithelial barrier integrity was measured by a combined metric of ZO-I expression, basolateral transfer of FITC-dextran, and *Spn* bacterial translocation from the apical to basal chamber of the transwell.

I hope this fully addresses reviewer’s concern.

The manuscript has been revised to add the findings from the new experiments. The revision refers to the **result** (page 10-11, line 233-238, 248-259), **discussion** (page 14-15, line 336-345), **method** (page 19-20, line 451-474), and **legends** (page 30, line 682-700).

**2.** In Fig 5E, is the amount of leakage seen in the mock sample normal? Would ALI cultures on D21 have less leakage because they have more ZO-1/tight junctions? Did the authors measure the TEER of their transwells?

**Author Response.** Thank you for the comment. In our past experiments with un-differentiated epithelial cells (day 7 ALI) and the new experiments (Fig. 5 revised manuscript) with differentiated epithelial cells (day 21 ALI culture), we observed significant leakage of FITC-dextran even in mock samples. We did not measure TEER of the transwell. However, we added another parameter of epithelial barrier integrity by measuring *Spn* bacterial translocation from the apical to basal chamber of the transwell. Because damaged epithelial barrier is a risk for *Spn* invasion, we modeled this by infecting day 21 differentiated epithelial cells (mock or IAV/rIL-17A treated) by *Spn* in the MOI of 1. The transwell media were then plated on blood agar plates to enumerate *Spn* colonies as a measure of bacterial translocation across damaged epithelia. Our data show the higher number of *Spn* bacteria were recovered from IAV+rIL-17A treated epithelial cells, suggesting that IL-17A treatment exacerbate IAV induced epithelial injury and barrier disruption, resulting in leaky epithelia that facilitate Spn translocation across damaged epithelia.

The manuscript has been revised to add the findings from the new experiments. The revision refers to Fig. 5, the **result** (page 10-11, line 233-238, line 248-259), **discussion** (page 14-15, line 336-345), **method** (page 19-20, line 459-474), and **legends** (page 30, line 694-696) sections.

**3.** What is the reasoning behind using PR8, a mouse adapted IAV, in the ALI model, which is composed of human NP epithelial cells? Fig 5F shows low levels of replication. Would using a human IAV that replicates better in human cells demonstrate better fold changes between infection, IL-17A treatment and the combination?

**Author Response.** We appreciate the comment from the reviewer. While PR8 is a mouse adapted influenza strain, we have consistently seen that PR8 can infect human nasopharyngeal epithelial cells in ALI epithelial model. Nonetheless, to address reviewer’s concern, the ALI experiments were repeated using a human IAV strain (see response above).

The revision refers to Fig. 5 and the modifications cited above (responses to points 1 and 2 from reviewer 2) applies to this section as well.

**4.** In the in vitro model, would the damage caused by infection or IL-17A treatment be significant enough to facilitate enhanced replication of Spn?

**Author Response.** Because damaged epithelial barrier is a risk for *Spn* bacterial invasion, we compared *Spn* translocation rate in differentiated epithelial cells (day 21 ALI). Mock or IAV infected/rIL-17A treated epithelial cells were infected with *Spn* in the MOI of 1 for 3 hours. The transwell media were then plated on blood agar plates to enumerate *Spn* colonies as a measure of bacterial translocation across damaged epithelium, the following day. Our data show the higher number of *Spn* bacteria were recovered from IAV+IL-17A treated epithelial cells, suggesting that IL-17A treatment exacerbate IAV viral-induced epithelial injury and barrier disruption, resulting in leaky epithelia that facilitate Spn translocation from top to bottom trans-well chamber.

The manuscript has been revised to add the findings from the new experiments. The revision refers to Fig. 5, the **result** (page 10-11, line 237-238, line 248-259), **discussion** (page 14-15, line 336-345), **method** (page 19-20, line 461-462, 470-474), and **legends** (page 30, line 695-697) sections.

**Reviewer #3:**

**1.** Their murine model only uses a single time-point to study to look at the inflammation associated with IAV infection. As they point out, the role of IL17RA has not been examined in the context of IAV infection, and it would be pertinent to look at the kinetics of the IAV infection/inflammation in the NP with earlier and later time points in both WT and KO mice. Do the KO mice always have lower inflammation, or do they eventually catch up to the WT-infected mice?

**Author Response.** We appreciate the important comment from the reviewer. The data in the original manuscript was from 7 dpi, which is usually the peak of IAV inflammation. To address the reviewer’s concern, we performed H&E staining to determine the differences in nasal inflammation between WT and IL-17RA KO mice on 3, 7, and 14 dpi. Further, we measured IAV viral load in the NP lavage on these three post-IAV infection time points (3, 7, and 14 dpi). Our data show that IAV induced low-grade inflammation at 3 dpi, and no difference in nasal inflammation was observed between IAV-infected WT and IL-17RA KO mice at 3 dpi. However, WT mice (compared to IL-17RA KO) had significantly higher inflammation at 7 dpi. Both groups (WT and IL-17RA KO) largely resolved nasal inflammation by 14 dpi. WT mice (compared to KO) had a higher viral load in the NP lavage on 3 and 7 dpi. However, no detectable viral load was found in the nasal lavage of WT and KO mice by 14 dpi. These data suggest that IL-17RA induced pathologic effect is largely limited during the peak inflammation (7 dpi), and the presence of IL-17RA doesn’t produce long-term defects in the resolution of inflammation or IAV viral control in WT mice.

The representative images under Fig. 2 have been replaced with new histology and viral load data. The revision refers to Fig. 2, the **result** (page 6-7, line 136-155), **discussion** (page 12, line 287-291), **method** (page 17, line 399-410), and **legends** (page 28, line 641-647) sections.

**2.** Even though this reviewer found their RNA-seq study fascinating with interesting results, they almost feel like they are tacked on to the study, as there is no follow-up on the results they obtained. Transcript levels also might not correlate with protein levels in the NP. It would help if they looked downstream, for example, RIG-1 or the NF-kappa B signaling pathway, and looked at protein levels as they did in Figure 1.

**Author Response.** We appreciate the comment from the reviewer. To address the concern, we purified nasopharyngeal epithelial cells from WT and IL-17RA KO mice at 7 dpi. Based on the information from RNAseq data, we performed western blot against significantly changing cellular markers between WT and IL-17RA KO mice. Our data show that epithelial cells from WT mice had higher protein expression of several key inflammatory mediators, such as pSTAT1, SOCS1, SOCS3, IRF1, and IRF2. These data support RNAseq findings that epithelial cells from IL-17RA KO mice had reduced inflammatory response.

**Additional note.** Because of the low cell recovery and lower protein yield, we had to pool nasopharyngeal epithelial cells from several experiments to recover enough protein for western blot experiment (to address reviewer’s concern). Therefore, out of several markers (inflammatory regulators) tried, the ones presented were the only markers that we could obtain meaningful signals. In our experience so far, performing western blots from nasopharyngeal epithelial cells is an incredibly cumbersome exercise. Nonetheless, we believe that the western blot data provided under Fig. 3 is meaningful enough to support RNAseq findings.

The revision refers to Fig. 3F, the **result** (page 8, line 187-192), **method** (page 20-21, line 487-494), and **legends** (page 28-29, line 650-652, line 664-675) sections.

3. The differences observed in membrane permeability are minimal in Figure 5E. They only show a one-time point at 2 hrs. Doing a kinetic run to determine how membrane permeability is impacted over time would be appropriate.

**Author Response.** Thank you for the comment. To address the concern from the reviewer 2, we repeated ALI experiments with differentiated (ciliated) human nasopharyngeal epithelial cells. In addition to the FITC dextran permeabilization assay, we used additional parameter, i.e.., *Spn* bacterial translocation assay to complement the findings from FITC-dextran experiment. Based on our initial preliminary experiments, the two hours of FITC-dextran treatment window was chosen because of the maximum leakage of FITC dextran at this time point. Therefore, we adhered to the same time window in our new ALI experiments with differentiated epithelial cells.

The revision refers to new data with differentiated epithelial cells (day 21 ALI) as Fig. 5, **result** (page 10-11, line 233-238, line 243-244), **discussion** (page 14-15, line 339-345), the **method** (page 19-20, line 459-470), and **legends** (page 30, line 694-695) sections.

**Part III – Minor Issues: Editorial and Data Presentation Modifications**

Please use this section for editorial suggestions as well as relatively minor modifications of existing data that would enhance clarity.

**Reviewer #1:** 1. The figure callouts in the text for Figure 5 were not always clear. For example, the FITC dextran data is referred to several times, but was never called out as Fig 5E in the text. Consider being more thorough with figure callouts to make referring back more straightforward.

**Author Response.** Thank you. All figures, including FITC dextran have been appropriately cited thorought the text.

2. Is there a statistic missing from figure 5A (IL-1b)? There are no statistical comparisons shown for the PR8+IL17-A condition.

**Author Response.** The Fig. 5 has been replaced with new data sets using differentiated NP epithelial cells infected with a human IAV.

3. Did the authors block Fc receptors in their antibody staining for flow? The methods in general are a bit thin. It would be helpful to increase the level of detail throughout.

**Author Response.** Thank you. The Fc receptor blocking was performed in all flow cytometry experiments. The flow cytometry method section has been revised to include Fc block information. (Page 16, line 387-389)

4. Line 90, should be 10ul instead of 10ml.

**Author Response.** Thank you. Addressed.

**Reviewer #2:** Please define what is IL-17RA at the beginning.

**Author Response.** Thank you. Addressed. Refereed to abstract section, page 2, line 33.

Can the authors provide some context for the reader as to the significance of looking at levels of IL-6, TNF-a and TGF-b?

**Author Response.** Thank you. Additional explanation has been provided to bring clarity on why the levels of IL-6, TNF-a and TGF-b were investigated.

The modifications refer to result (Page 6, line 126) and discussion section (Page 13, 14 and line 307-311 and line 323-331)

90 should probably read 10uL as the low volume.

**Author Response.** Thank you. Addressed.

In Fig 4, the authors are comparing D7 vs D21, which is hard since the y-axis are different in some panels. Please include on the same graph for easier comparison.

I would be helpful to the reader to include a model.

**Author Response.** To address the reviewer’s concern, the prior ALI data in the original submission has been replaced with new data sets using differentiated epithelial cells. The graphs in Fig. 5 have been reworked to clarify the presented data.

**Reviewer #3:** 1. In Figure 3, they show lower levels of S. pneumoniae levels in the NP (KO vs. WT) correlate with lower levels in the blood. They should normalize their levels of bacteria in the blood to what is present in the NP, as that would be a more accurate comparison.

**Author Response.** Thank you. We agree with reviewer’s comment. Since we already revised the figure by following reviewer 1’s suggestion to present the blood bacterial data in log scale, we provided the figure below to address the reviewer’s concern. The result indicates that the KO mice had reduced bacterial burden in the blood by comparing to WT with the NP normalization, supporting the data presented under Fig. 4A.

2. The strain of Spn used is only mentioned in one of the figure legends. It would be appropriate to describe the strain in the method section and the reference if available.

**Author Response.** Thank you. The *Spn* strain has been added to the method section. In addition, the strain name is included in all figure legends.

3. In line 90, the volume for infection with IAV should be ul and not mL.

**Author Response.** Thank you. Addressed.

4. Treatment with IL17A should say recombinant.

**Author Response.** Thank you. Addressed.

5. In Figure 5, an extra hyphen is added to IL-17A with treatment with PR8.

**Author Response.** Thank you. The Fig 5 has been replaced with new figure.

**Final note from Authors.** In addition to above modifications, the manuscript has been revised for any minor error or inconsistency.

PLOS authors have the option to publish the peer review history of their article (what does this mean?). If published, this will include your full peer review and any attached files.

If you choose “no”, your identity will remain anonymous but your review may still be made public.

Do you want your identity to be public for this peer review? For information about this choice, including consent withdrawal, please see our Privacy Policy.

Reviewer #1: No

Reviewer #2: No

Reviewer #3: No

Figure Files:

While revising your submission, please upload your figure files to the Preflight Analysis and Conversion Engine (PACE) digital diagnostic tool, https://pacev2.apexcovantage.com. PACE helps ensure that figures meet PLOS requirements. To use PACE, you must first register as a user. Then, login and navigate to the UPLOAD tab, where you will find detailed instructions on how to use the tool. If you encounter any issues or have any questions when using PACE, please email us at figures@plos.org.

Data Requirements:

Please note that, as a condition of publication, PLOS' data policy requires that you make available all data used to draw the conclusions outlined in your manuscript. Data must be deposited in an appropriate repository, included within the body of the manuscript, or uploaded as supporting information. This includes all numerical values that were used to generate graphs, histograms etc.. For an example see here on PLOS Biology:

http://www.plosbiology.org/article/info%3Adoi%2F10.1371%2Fjournal.pbio.1001908#s5.

Reproducibility:

To enhance the reproducibility of your results, we recommend that you deposit your laboratory protocols in protocols.io, where a protocol can be assigned its own identifier (DOI) such that it can be cited independently in the future. Additionally, PLOS ONE offers an option to publish peer-reviewed clinical study protocols. Read more information on sharing protocols at https://plos.org/protocols?utm_medium=editorial-email&utm_source=authorletters&utm_campaign=protocols
